# From biobank and data silos into a data commons: convergence to support translational medicine

**DOI:** 10.1186/s12967-021-03147-z

**Published:** 2021-12-04

**Authors:** Rebecca Asiimwe, Stephanie Lam, Samuel Leung, Shanzhao Wang, Rachel Wan, Anna Tinker, Jessica N. McAlpine, Michelle M. M. Woo, David G. Huntsman, Aline Talhouk

**Affiliations:** 1 Department of Molecular Oncology, BC Cancer Research Centre, 675 West 10th Avenue, Vancouver, BC V5Z 1L3 Canada; 2 BC Children’s Hospital Research Institute, 938 West 28th Avenue, Vancouver, BC V5Z 4H4 Canada; 3 Department of Pathology and Laboratory Medicine, Faculty of Medicine, University of British Columbia, 2211 Wesbrook Mall, Vancouver, BC V6T 2B5 Canada; 4 Department of Medicine, Faculty of Medicine, Division of Medical Oncology, University of British Columbia, 2775 Laurel Street, Vancouver, BC V5Z 1M9 Canada; 5 BC Cancer, 600 West 10th Avenue, Vancouver CentreVancouver, BC V5Z 4E6 Canada; 6 Division of Gynecologic Oncology, Department of Obstetrics and Gynecology, University of British Columbia, Vancouver, Canada; 7 OVCARE Research Program, Vancouver, Canada; 8 Present Address: Division of Gynecologic Oncology, Department of Obstetrics and Gynecology, University of British Columbia, 5th Floor (593), 828 West 10th Ave, Vancouver, BC V5Z 1M9 Canada

**Keywords:** Biobanks, Biospecimens, Biobank-technologies, Precision medicine, Data commons, Laboratory Information Management Systems (LIMS), Federated systems, Data governance

## Abstract

**Background:**

To drive translational medicine, modern day biobanks need to integrate with other sources of data (clinical, genomics) to support novel data-intensive research. Currently, vast amounts of research and clinical data remain in silos, held and managed by individual researchers, operating under different standards and governance structures; a framework that impedes sharing and effective use of data. In this article, we describe the journey of British Columbia’s Gynecological Cancer Research Program (OVCARE) in moving a traditional tumour biobank, outcomes unit, and a collection of data silos, into an integrated data commons to support data standardization and resource sharing under collaborative governance, as a means of providing the gynecologic cancer research community in British Columbia access to tissue samples and associated clinical and molecular data from thousands of patients.

**Results:**

Through several engagements with stakeholders from various research institutions within our research community, we identified priorities and assessed infrastructure needs required to optimize and support data collections, storage and sharing, under three main research domains: (1) biospecimen collections, (2) molecular and genomics data, and (3) clinical data. We further built a governance model and a resource portal to implement protocols and standard operating procedures for seamless collections, management and governance of interoperable data, making genomic, and clinical data available to the broader research community.

**Conclusions:**

Proper infrastructures for data collection, sharing and governance is a translational research imperative. We have consolidated our data holdings into a data commons, along with standardized operating procedures to meet research and ethics requirements of the gynecologic cancer community in British Columbia. The developed infrastructure brings together, diverse data, computing frameworks, as well as tools and applications for managing, analyzing, and sharing data. Our data commons bridges data access gaps and barriers to precision medicine and approaches for diagnostics, treatment and prevention of gynecological cancers, by providing access to large datasets required for data-intensive science.

**Supplementary Information:**

The online version contains supplementary material available at 10.1186/s12967-021-03147-z.

## Background

The collection, storage, management, and distribution of human biospecimen for diagnostic pathology [1–3] can be traced as far back as the 1900s [3]. To meet research needs in the postgenomic era, modern day biorepositories [4] support scientists to derive disease-specific insights [5] by aiding the investigation of genetic underpinnings [6–8], elucidating etiology, and evaluating disease progression and therapeutic response; they are the backbone of precision medicine [9, 10], biomedical, and translational research [1, 2, 11].

The last decade has seen advances in biotechnology such as next generation sequencing (NGS), and the emergence of “omics” techniques for precision medicine (e.g., genomics, transcriptomics, proteomics, metabolomics, and epigenomics). These innovations coincided with breakthroughs in computing, artificial intelligence (AI) and analytics, enabling discrimination between disease with greater precision [12]. This has created an unprecedented demand for high quality biospecimens and associated data, including clinical, molecular, imaging, and other types of data generated during research [11]. Innovations in database cloud storage and computing infrastructures to support data intensive science have further contributed to revolutionizing resources available to address modern research needs [13, 14]. As federated models for aggregating data and biomaterials have emerged as favoured approaches for identifying enough patients with specific clinical or molecular features, the importance of interoperability between biobanks and related databases has been accentuated [4, 7, 15]. Specimen collections have become virtual [13], flexible and interoperable, hosted on internationally harmonized infrastructures [7] and optimized for secondary research [7, 13]. Present day research environments and needs have led to the development and implementation of *data commons* [16, 17]*,* bringing together, within a research community, diverse data, computing infrastructure, as well as tools and applications for managing, analyzing, and sharing interoperable data. This has created an opportunity to maximize collaborations and to extend the value generated from primary data collection [18].

In 2016, as part of BC’s multidisciplinary gynecological cancer research team (OVCARE), we undertook a comprehensive review of the landscape of the data assets available within our local research environment and assessed the infrastructure needs required to support data storage and sharing within our research community. Herein, we describe the roadmap undertaken for the creation of a data commons, transforming a traditional tumour biobank and a collection of data silos, into an integrated and comprehensive infrastructure to support current and future research needs of an expanding team.

## Results

### Matching technical solutions to research needs

OVCARE started in 2000 as an initiative between the British Columbia Cancer Agency, the University of British Columbia and the Vancouver Coastal Health Research Institute to accelerate research discoveries and translation to the clinical settings and to improve the lives of women with ovarian cancer or those at risk. Today, OVCARE is an internationally recognized multidisciplinary team of physicians and scientists who are breaking new grounds in improving diagnosis, prevention and treatment of all gynecological cancers [19–27].

OVCARE’s research has been powered by the gynecological tumour bank and the Cheryl Brown Gynecological Cancers Outcomes Unit. Through the course of research, a plethora of molecular and genomic data was historically held by researchers that generated them. Similarly, clinical data from chart reviews are obtained to support clinical studies and held with clinicians. These data were in incompatible formats that needed significant manual manipulation and curation to be integrated. Moreover, each collection was governed by different ethics agreements that restricted the use of data and kept it in silos. This was becoming a barrier to novel data-intensive research, requiring the integration of multiple data sources; undertaking such projects was challenging, time consuming, and prone to errors; the OVCARE leadership recognized that current research needs were not being met through existing infrastructure.

A broad stakeholder engagement effort in 2016 kicked off with the objective to work with researchers, clinicians, scientists, and technicians at various institutions, to map out a collective future vision, identifying research needs, and re-thinking present infrastructure. Engagements with key stakeholders identified research priorities which were expanded into a list of fundamental requirements (Table 1) relevant to the collection and optimization of biospecimen, clinical, and molecular/genomics data, as well as a governance model of the resulting infrastructure. In addition to generating efficiency, limiting errors and honoring patient consent, fundamental research requirements included the maximization of secondary use of data, that enables data collected for one purpose, to be used in a completely different context. For example, chemotherapy drugs dispensed at our pharmacy are collected for administrative purposes (billing) but can also be used to link with patient phenotype, genotype, and outcome to investigate which patients benefit from these therapies more than others. Another important need was to generate novel research hypotheses by considering simultaneously various data that could never before be considered at the same time. Patterns that may not have been obvious previously may emerge to drive future innovative research. Another important need was to use translational studies to help inform patient care, as well as use data generated from patient care to ask new research questions to continuously try to better fill gaps in understanding of disease etiology and progression. In upcoming sections, we further describe more of these requirements in greater detail.

**Table 1:** Summary of fundamental research and infrastructural needs of OVCARE’s research community

	**Fundamental research requirements**
1	Generate efficiencies in data collection, storage and analysis to maximize utility of collected data
2	Limit errors in data handling and ensure reproducibility of research findings
3	Protect patients’ privacy and honor their consent
4	Optimize secondary and continuous use of data generated from research and clinical care
5	Facilitate the recruitment of patients in various clinical studies
6	Identify specimen from patients with specific clinical, molecular and genomic characteristics
7	Integration of medical and clinical data with molecular information to enable the discovery and testing of new associations and hypotheses towards translational research
8	Organize data towards a learning healthcare system where translation is bi-directional: Evidence-based research is used to inform practice, and the data generated during clinical care is in turn used to inform guidelines, generate hypotheses and trigger pragmatic trials
	**Functional and infrastructural IT requirements**
1	Allow batch data imports and exports
2	Facilitate validation of data entered to minimize errors (e.g. returning an error message when text is entered instead of a numeric value)
3	Easy-to-use and customizable user interfaces
4	Support both prospective and retrospective data collection mechanisms
5	Adapt to changing needs between studies and projects, as well as over time
6	Track biospecimen locations, usage and shipment to both local and offsite storage locations
7	Support multi-tenancy for the banking of biospecimens from distributed and diverse studies lead by different investigators interested in sharing resources
8	Adherence to best practices in privacy and security, such as, support for data encryption, audit trails on all user actions and data changes for regulatory compliance, configurable user privileges, role-based access control and adherence to federal regulations with respect to deidentification of specimen and tracking of consent
9	Support interoperability and integration with other institutions, systems, and data sources to facilitate data sharing
10	Potential to scale-up biospecimen and user capacity at no added cost
11	Stable and mature vendor and community support

#### Biospecimen collection

OVCARE employs two models for biospecimen recruitment: the first is a general banking model, with broad scientific aims, and where specimens are obtained from consented participants and stored until needed. The second is a study-based banking model, where participants are recruited to address specific study aims, with a pre-defined protocol and pre-planned specimen collection. To accommodate both approaches, the biorepository infrastructure needed to manage accrual of specimen in a patient-centric approach, retain the context of the patient’s clinical history, as well as support basic biospecimen collection, storage, and distribution across multiple studies at different sites, under both recruitment models. This includes inventory control, the ability to track sample availability and location, as well as track generated derivatives (e.g., xenografts and organoids). The infrastructure needed to be adaptable to changing needs between studies, projects, as well as over time, with the ability to preserve the natural history of the data. Access control that varied for different user-groups was a critical feature to enable adherence to regulatory requirements and health research best practices. Data security, deidentification of specimens and tracking of consent were also important for the same reasons, in addition to the need to operate and manage the biorepository with minimal support from institutional and research IT.

**Fig. 1:**
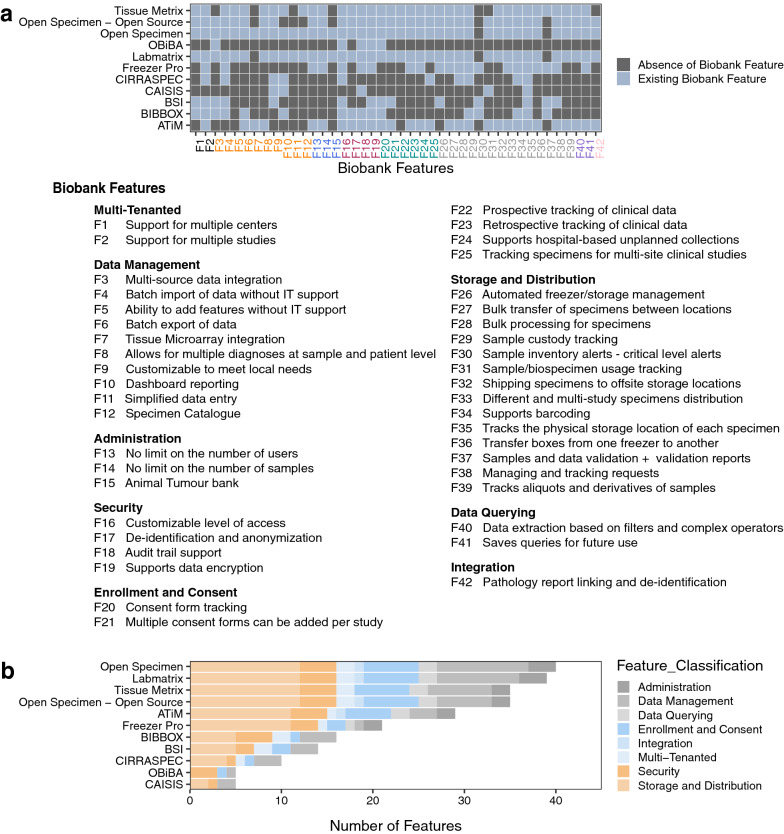
Needs-to-biobank mapping and the number of requirements fulfilled by each LIMS. **a** Tiled plot of the mapping of each biospecimen research need to the biobank solution meeting that need. Surveyed biobanks are plotted on the y-axis and research needs (desired biobank features) are plotted on the x-axis, grouped and colored by feature class. **b** Barplot on the overall number of features provided by a specific LIMS. The LIMS solutions are plotted on the y-axis and the number of features provided are plotted on the x-axis

We compiled a comprehensive list of requirements (Additional file 2: Table S1) from our stakeholder meetings and we used it to guide our scan of the landscape of existing laboratory information management systems (LIMS) (Additional file 2: Table S2—S11, and Fig. 1). This resulted in the identification of OpenSpecimen [28], a LIMS based on caTissue [29], a mature system with over 15 years of use by the research community. OpenSpecimen addressed more requirements from our list in comparison to other options we considered. It is an open-source software with commercial support, in use by over 70 biobanks across 20 countries. The commercial support ensures ongoing software testing, updating, and continuous improvement. This is in addition to the availability of technical support, and access to a community of experienced users through active forums.

In this LIMS, biospecimens can be processed individually or in bulk, with rapid barcode-based scanning available to enter information on multiple patient samples at once. This enabled high throughput processing and efficient migration from our legacy LIMS. Options for data annotation and storage management allowed us to optimize specimen storage, a costly resource in our research community (e.g., − 80 freezers) [29].

The OpenSpecimen LIMS enabled customization of data entry forms via a graphical user interface (web interface) to match study-specific needs without requiring software development. The platform met most of our IT requirements as it supported role-based access control and provided an audit trail of every user operation [30]. The system was also easy to use with graphics-based queries that enable searching for stored data about participants, biospecimen, or projects, without requiring any programming, making the moderately complex queries accessible to most users. Queries could also be performed via REST API (Representational State Transfer Application Programming Interface) using a SQL (Structured Query Language)-like query language. This facilitated automation of data downloads for analytics pipelines through the incorporation of query scripts.

The system enables standalone plugins through a software development kit. These plugins can be made publicly available to the community. For example, the tissue microarray (TMA) plugin can manage TMAs on OpenSpecimen by linking to donor blocks and describing details of experiments done on the different slices of the TMA blocks. Finally, the interoperability with other systems was important to expand linkage within the data commons. The vendor provides integration with electronic data capture applications (REDCap, Open Clinica), electronic medical record systems (EPIC, Velos), pathology systems (CoPath, Cerner, Aperio), as well as Health Level Seven (HL7) messages; a capability which can further support inclusion of participant and biospecimen information from distributed systems.

#### Molecular and genomics data

Various molecular and genomics data are generated through the course of research. These include next generation sequencing, proteomics, gene expression, targeted sequencing, as well as immunohistochemical data. These data were primarily generated to answer specific research hypotheses and were supported by public, government, and philanthropic funds, with an implicit obligation to minimize duplication of efforts and to optimize their secondary use in later research. The ability to consider all this data simultaneously can uncover novel patterns, trends, and unknown correlations. This may prompt new hypotheses and spark new insights into novel research directions. To achieve this level of integration, we would need to track which analytical assay was performed on which samples and link back to those data. To facilitate the interrogation of this complex data, an exploration tool was needed to visualize resulting multidimensional datasets and simultaneously investigate molecular profiles and clinical attributes.

We adopted the cBioPortal for Cancer Genomics [31], one of the most recommended and widely used [32–36] pan-cancer analytics web tools to facilitate interactive exploration, mining, analysis, and visualization of multidimensional datasets derived from tumor samples collected from various cancer studies [31, 37]. Developed at the Memorial Sloan Kettering Cancer Center (MSK), this platform is used by large cancer genomic studies (TCGA [38], TARGET [39]), and publicly available data can be downloaded and queried alongside our own collections.

The cBioPortal enables the collection of various genomic data on each tumor sample, including non-synonymous mutations, copy-number alterations (CNAs), mRNA and microRNA expression data, DNA methylation data, protein, and phosphoprotein level data [31]. Each of these data types is integrated and stored at the gene level to allow investigators to probe for the presence of specific biological events (e.g., gene mutations, deletions, amplifications, and expression levels in each sample) [37], and compare discrete genomic events and patterns across samples and across multiple integrated data types [31]. Stored gene-level data is integrated with de-identified clinical data to probe patient clinical outcomes to support the development or testing of hypotheses on frequently altered genes in specific cancers [31, 37]. In addition, it enables the investigation of the prognostic roles of certain genes in gynecological and other cancers [34], correlations between mutations, expression profiles, clinicopathological features, and potential diagnostic and therapeutic targets for certain cancer types.

#### Clinical data

Clinical data at OVCARE are obtained and collected for the purpose of evaluation of outcomes, improvement of the quality of patient care, as well as for research. Some of these data were historically managed by the Cheryl Brown Outcomes Unit for the purpose of outcomes research on ovarian cancer patients referred to BC Cancer, the provincial tertiary cancer center. The BC Cancer Registry provided the Cheryl Brown Outcomes Unit regular data updates such as the identification of patients with cancer and their vital statistics, which were supplemented by exhaustive chart reviews. In addition to the Cheryl Brown Outcomes Unit, clinicians often conducted chart reviews for other clinical studies; the resulting data was held separately. In 2016, the scope of data collection at the Cheryl Brown Outcomes Unit was limited to ovarian cancer and did not take full advantage of other available data. Collecting clinical data was resource-intensive and the effort needed was not sustainable in the long run. Moreover, the mandate of the Cheryl Brown Outcomes Unit expanded to enable OVCARE’s researchers to study all gynecological cancers in the province of BC, especially those cancers that do not require referral to a cancer center (e.g., in BC, up to 50% of patients with endometrial cancer are treated by gynecologists in their communities). Thus, an important priority for the team was to create efficiencies in clinical data collection and to standardize, integrate, and link all gynecologic cancer clinical data from various sources and consolidate clinical data in a single database. This would allow researchers to understand what clinical data is already available, thereby streamlining their own data collection strategies which would, in turn, directly contribute to a master database. To maximize the re-use of clinical data, standardization of ontologies across projects was needed, as well as the creation of infrastructure to serve as permanent storage with an easy-to-use data collection interface adaptable to fit the needs of various research projects. This would allow standardization of data collection, to the extent possible, and minimization of errors. Consequently, this would improve the overall quality of data, maximize interoperability and reusability, and optimize data analysis. Management of sensitive clinical data requires security, privacy and the use of tools and technology with institutional approval. We also needed rigorous security and privacy measures, and comprehensive audit trails for tracking data manipulation, exports, and downloads for both single and multi-centered research studies, including tracking data access.

To support OVCARE’s clinical data requirements, we adopted Research Electronic Data Capture (REDCap), a widely used, free and flexible web-based application [40, 41] developed at Vanderbilt University for clinical and translational research. It is one of the most popular research electronic data systems implemented in 141 countries by over 1,000,000 [42] studies, including our institutions. REDCap’s flexible design supports permanent database collections which can be augmented by both patient/study-centric surveys or data collection forms, and includes a rich set of modules that support today’s diverse and multi-scaled biomedical research operations [41].

#### Governance structure

To manage the various integrated datasets (biospecimen, molecular, genomic, and clinical data) we needed to ensure proper governance, protocols, and standard operating procedures to support data sharing, streamline data requests and inquiries, undertake scientific review or requests, and ensure availability of ethics approval. We envisioned a single portal application for all requests and queries with a backend database keeping track of details of requesting researchers, description of projects, required resources as well as their associated ethics application and certificates of approval. This infrastructure would facilitate compliance with ethics and maintain a log of all activities.

We adopted Oracle Application Express (APEX) [43]**,** by the Oracle Corporation**,** to develop this portal application. Already available at our institution, APEX, is a low-code, data-driven platform for rapid development and deployment of scalable and secure web applications. Applications are implemented in a preconfigured environment; all development was done through a web interface that is mostly GUI (graphical user interface)–based. The middle-tier functions of the web application software stack, such as parsing Hypertext Transfer Protocol (HTTP) requests and session management, are fully automated, and all operational aspects of the system (data backup, software patches and updates) are managed by institutional IT.

### Implementation

**Fig. 2:**
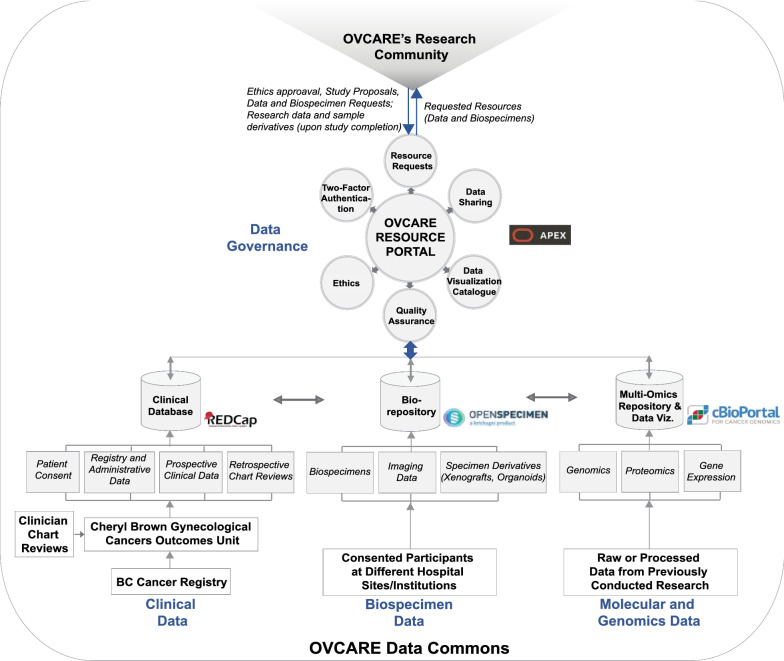
OVCARE’s data commons infrastructure and software stack. The overall data commons infrastructure comprises of five main components: (1) A clinical database (REDCap) that consolidates and manages clinical data collections from the BC Cancer Registry and the Cheryl Brown Gynecological Cancers Outcomes Unit, (2) a Library Information Management System (OpenSpecimen) that stores and manages biospecimens collected from consented participants at different hospital sites (i.e. Vancouver General Hospital, the University of British Columbia Hospital, BC Cancer Vancouver, and now a few more centers in BC, (3) the cBioPortal that supports the exploration, analysis and visualization of clinical attributes and molecular profiles from patient tumor samples, (4) the OVCARE Resource Portal (ORP) that governs data and resource sharing based on stipulated protocols, standard operating procedures and research ethics, and (5) the Research Community (this includes the OVCARE internal research and informatics team, and the broader research community that OVCARE serves). Each of the components (REDCap, OpenSpecimen, cBioPortal, ORP) identified to meet our research needs are separately hosted in our hospital’s computing environment and programmatically interlinked through API calls. The data from the different domains are interlinked using system-wide unique identifiers that link patients to their biospecimen collections and molecular/genomics data. To access the amassed clinical and biospecimen collections, authenticated researchers in the OVCARE research community send data and sample acquisition requests to the ORP through which those requests are met by informatics staff, if all stipulated requirements including ethics approval are met. Upon successful data and sample acquisition, researchers conduct their respective studies, and the data generated (raw or processed, and/ biospecimen derivatives) from their research are retuned to OVCARE making it available for re-purposing/secondary use. Furthermore, molecular data returned to the data commons are linked back to the available and stored patient biospecimens. Together with clinical outcomes, these molecular profiles are further explored, analyzed and visualized using the cBioPortal

**Fig. 3:**
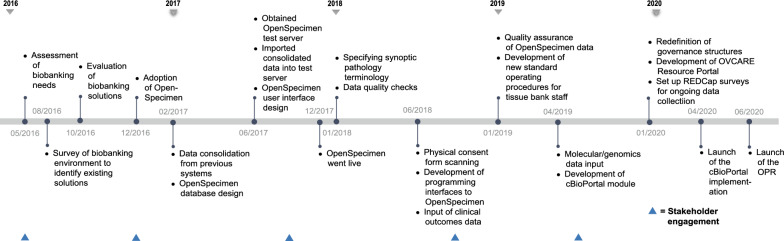
Implementation timeline of OVCARE’s data commons

The various components of the data commons infrastructure and software identified to meet the domain-specific needs described in the previous section are illustrated in (Fig. 2). This infrastructure is implemented behind institutional firewalls with only the resource portal accessible through the world wide web. The path to implementing this infrastructure was not linear and continues to evolve, despite the linear timeline presented in (Fig. 3).

In early 2017, we completed a survey of existing biobanking solutions to select one that provided the best fit to our needs at that time. In June 2017, a test server was obtained to run local instances of the selected LIMS, OpenSpecimen, to conduct functionality, integration, and unit testing of all components of this software. This enabled us to evaluate OpenSpecimen's features firsthand and to determine the required resources to operate the infrastructure with optimal performance in our current computing and research environment. We tested for performance and evaluated operation workflows by diverse types of users, both technical and nontechnical, to perform daily biobanking activities. We fully adopted OpenSpecimen in December of 2017. Following this migration, we worked with researchers to gather available genomic datasets and link their availability to the respective biospecimen in OpenSpecimen as well as indicate where data are held. As we continue to expand this resource, we will add availability of images of pathology slides, associated with each tumour block and link to them. To prototype the cBioportal integration, we gathered molecular data for one ovarian cancer subtype, collected from prior studies which were integrated with specimen availability and key clinical outcomes in cBioportal, using specimen ID. We recently launched this prototype and it is currently under evaluation.

**Fig. 4:**
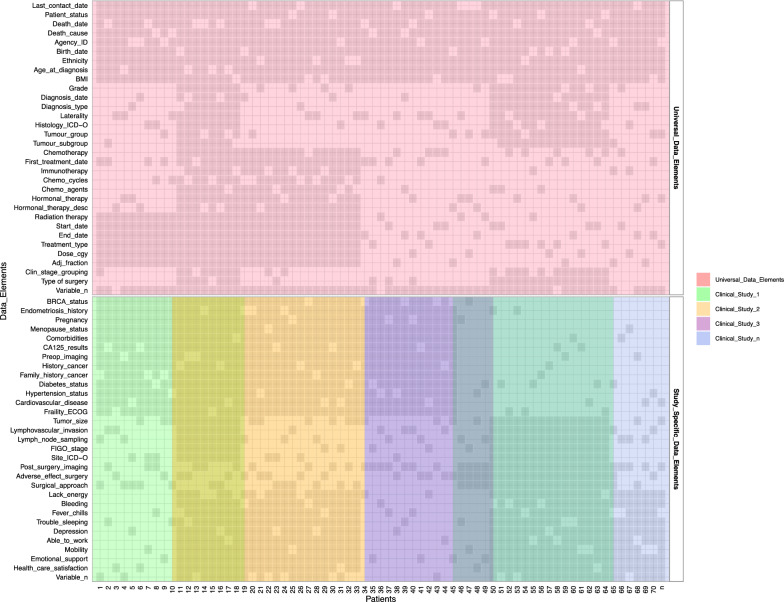
Clinical and outcome data on all gynecological cancer patients diagnosed in British Columbia. In the tiled plot, data elements (demographic, medical history, pathology, chemotherapy, radiation, surgery and quality of life data) were plotted on the y-axis against gynecological cancer patients (patient 1 to n) on the x-axis. Darker tiles indicate availability of data on a patient per data element. Clinical studies (study 1 to n) are interested in certain patients with available data on specific data elements. Subsets of patients overlap between clinical studies

For clinical data, we expanded the mandate of the Cheryl Brown Outcomes Unit to include clinical and outcome data on all gynecological cancer patients diagnosed in British Columbia. We also obtained ethics approval to permanently retain clinical and outcomes data from all clinical studies in our group. We maximized data we can receive from administrative sources, such as the BC Cancer Registry, as this provides access to clinical data for all patients and minimizes the need for broad chart reviews (Fig. 4). We included elements, such as the date of diagnosis, date of last clinical appointment, vital statistics, International Classification of Diseases (ICD)-10 morphology codes, tumour stage, and grade. We are presently investigating additional data, such as systemic therapy (chemotherapy and radiation therapy received). The second step of clinical data integration involved adding clinical studies with chart reviews. To enable that, we needed to map different data elements to unique concepts. This further facilitated the identification of variables that are of greatest interest to researchers in our group. We then developed consistent data definitions, standards, and semantics for each data element to ensure that all data can be integrated within the data commons. Future data collection will consult these data standards to ensure prospectively harmonized clinical data.

Finally, to manage all data assets and resources, we developed the OVCARE Resource Portal (ORP). Designed and customized to fit the needs of OVCARE users, this solution is implemented in the APEX software and launched in June 2020. This portal has helped to consolidate workflows and all data and resource requests, helping to ensure proper governance and compliance with protocols, standard operating procedures, and Research Ethics Board requirements.

Each of these implementations (REDCap, OpenSpecimen and cBioPortal) are hosted separately on the hospital’s research IT network and solely accessible to informatics staff. Only the resource portal is accessible for researchers to make requests. Data are integrated through unique identifiers that link the various tables from each database at the patient level or at the specimen level. Data linkage to fulfill various study requirements is done programmatically through API calls.

To request data, researchers create user accounts on the ORP, and if needed, associate the principal investigator profile to their account. Authenticated researchers can then submit information (study proposal, ethics approval and study requirements) on the study for which resources will be requested. A project reference number created for progress tracking is then issued to the researcher and an ORP-generated email sent to the informatics staff notifying them of a new study proposal. Received proposals are subsequently processed and sent for review and approval by a committee of reviewers selected from the OVCARE community, after which resource requests are fulfilled. Researchers return to the data commons any raw and processed data that results from their studies, as well as any derivatives produced by their research (cell lines, DNA extractions, organoids).

## Discussion

We have described the journey followed towards implementing a data commons to benefit the gynecologic cancer community in British Columbia. This infrastructure democratizes access to resources shared by the entire community and brings together the whole gynecological cancer community in BC to work towards a common goal: to reduce death and suffering for women with gynecologic malignancies. To safeguard our data assets and maximize their utility, we have created a unified infrastructure, along with standardized operating procedures to meet research and ethics needs. The core expertise in data management and informatics which was developed in this process generated efficiencies in data collection to maximize the value of data and stretch research funds by optimizing their secondary use. The proposed governance structure streamlines requests, ensures scientific integrity of projects, as well as adherence to privacy, security and ethical disclosure of patient-specific data.

Through our investigations we found that no single solution can meet all the different data needs. Rather, the integration of multiple solutions can help us achieve the desired outcome. While the software and technology stack used to implement the current infrastructure will serve us for the near future (5 years), the data storage and management field is moving at a very fast pace, and we may need to re-assess our requirements soon. In choosing our software stack, we needed to balance between risks associated with open-source and open-access which provided affordable solutions and more control, but where little support is available and software code could stop being maintained, versus going with a corporate software that provides more technical support and liability, but can be potentially very costly to set up and maintain. To mitigate this, we went with hybrid models where possible and selected software that had an active community of users and that enabled some degree of customization.

The data we collected as part of primary research or for administrative purposes needed to be harmonized for integration. For example, some data sources report “tumor grade” as “high or low”, while others report numeric grades: 1, 2, 3, 4; occasionally reported as “male and female”, gender could also be represented as “M and F”, “1 and 0” or “1 and 2” [44]. Integration of such data presents “unique technical, semantic, and ethical challenges” [45] and could also result in large amounts of unusable data due to loss in translation. Developing standards a priori streamlines semantics and ontologies, avoids data wastage, increases data quality, and supports effective data integration, sharing and reusability, while also saving significant time and costs required to pool, process and share data [44, 46]. Future efforts to connect with other biorepositories and similar databases from other centers rely on adopting standardized ontologies to facilitate data sharing. Policies for ensuring data quality and security were also defined, including, establishing team and user roles, and data access levels; ensuring that all processes from data acquisition to distribution are compliant to stipulated policies and research ethics.

The data commons is overseen by three principal investigators including an informatician, a medical oncologist, and a gynecological oncologist. The team that operationalizes this infrastructure includes a part-time database manager and a data scientist who work on various data integrations. A lab technician and a clinical coordinator with the help of various co-op students facilitate specimen acquisition, storage, as well as data collection. Occasional consultations with pathology and oncology fellows are needed.

Our team continues to curate and harmonize available data to maximize their utility. For example, in the next year, we will add digital pathology images as well as have the ability to upload our collection to data enclaves where it can be linked to other administrative data including health service utilization and prescription drugs. This will result in a very rich data ecosystem, which will be ripe for novel scientific discovery and can enable research never before possible.

In the very near future, we are expanding our data commons to make it more patient centric. We are launching an online consent process so that we can reach a broader patient population to invite them to participate in research. We are also adding patient reported outcomes (PRO) to the data commons.

## Conclusions

In contrast to traditional biorepositories, the consolidation of heterogeneous datasets and biospecimens from various distributed systems, clinical studies, and research institutions, into a data commons presents important opportunities to drive translational medicine. A seamless data environment for clinical and research data can be achieved through shared policies and technologies, and privacy-preserving open computer architectures and storage platforms.

The success and sustainability of data commons rely first and foremost on fostering a scientific community capable of using the open and connected data environment. Secondly, the appropriate technological solution suitable for each type of data needs to be in place; there is no single solution that can be adapted to all data collections but multiple solutions should be integrated. Lastly, the proper governance structure is needed to grapple with the unique challenges presented in cross-institutional and multi-disciplinary research, resource integration, data sharing and data harmonization for greater interoperability.

In this paper, we present methods developed and applied to successfully establish a federated and scalable infrastructure that extends OVCARE’s traditional tumour biobank, outcomes unit and a collection of data silos, into an integrated data commons. To this end, we gathered and analyzed all research requirements of participating institutions under three main domains: (1) biospecimen collections, (2) molecular and genomics data, and (3) clinical data, and identified, developed, and implemented solutions that meet each of these requirements. We further built a governance model and a resource portal to effectuate protocols and standard operating procedures, to support data and biomaterials aggregation, sharing, harmonization and governance, across all participating institutions. We believe such infrastructures will help break barriers to the access of large datasets required to elucidate and improve our understanding of complex and rare diseases, providing powerful opportunities for knowledge discovery and translation towards improved patient care.

## Methods

### Needs assessment

To identify research needs and gather infrastructural requirements, stakeholders were engaged from all participating institutions. Discussions and one-on-one meetings with individual researchers, as well as brainstorming meetings to map out general research direction and requirements for the upcoming 5–10 years were held. Further discussions were conducted with institutional research IT to understand security, data management and sustainability requirements. Identified direction and priorities were expanded into a list of requirements (Additional file 2: Table S1) relevant to the collection and optimization of biospecimen, clinical, and molecular/genomics data, as well as a governance model of the resulting infrastructure.

### Technical solutions

For each of the domain-specific requirements (governance, biospecimen, clinical and molecular/genomics data), technical solutions were identified to meet the needs established under that domain. Solutions required for managing clinical and molecular/genomics data (REDCap and cBioPortal respectively) were previously well established, tested, implemented, and proven to meet the needs emerging from these two data domains in our research environment.

To identify a LIMS solution that met all/most of the identified biospecimen requirements, we surveyed the biorepository and LIMS environment (Additional file 1) and identified nine prominent software solutions that we comparatively evaluated. Based on publications and online documentation, we collected and analysed data on all identified biobanking software and examined the features and functionality of each with respect to our requirements (Additional file 2: Table S12). We also conducted meetings, interviews, and live interactive demos with various software vendors. A list of features per identified platform (Additional file 2: Table S2—S11) was generated to which each of our requirements was considered to identify the solution that best addressed our needs (Additional file 2: Table S12). Results from this survey were presented in a second stakeholder meeting where we discussed the suitability and utility of the identified LIMs, and decided to further evaluate OpenSpecimen.

Based on collected biospecimen data, we defined database concepts (entities, attributes, relationships, and constraints) and customized the backend OpenSpecimen database (running MySQL). We obtained a test server (implemented in Java and Apache Tomcat) and installed a Linux-based local instance of OpenSpecimen in our computing environment. During these pilot runs, frequent inquiries were made with software vendors on features, components, integration, and interoperability functions, including the identification of missing requirements. Following successful tests, data from legacy systems was then consolidated into the server by leveraging OpenSpecimen’s batch uploads utility. We further designed and developed the user interface and configured and customized OpenSpecimen to our unique requirements before moving it into production.

### Data standardization and integration

The vision of modern translational medicine largely hinges on the integration of large-scale clinical and molecular profiles of patients to derive hypotheses and novel insights into a patient’s disease [45, 47, 48]. The data at OVCARE is derived from multiple disparate sources. To consolidate data from several databases, we began rigorous data validation and quality control checks. We extensively reviewed all biospecimen data, which included: (1) checking, locating and uploading all physical consent forms to ensure a digital record in our database, (2) uploading all physical biospecimen requisition forms, (3) reviewing all pathology diagnosis (by pathologists with gynecological subspecialty), and (4) locating and confirming availability of all specimens. The process of integrating molecular and genomics datasets into OpenSpecimen required close collaboration with researchers with expertise in the interpretation of these data. At the start of 2019, we obtained and consolidated from all OVCARE researchers any previously collected “-omics” datasets. As a first step, we mapped the omics data back to specimen and created tags indicating their availability in OpenSpecimen patient profiles. The second step of this process started in April 2020 with the implementation of cBioPortal for data visualization and analytics.

To consolidate clinical data, we derived a two-step approach whereby we use a minimal set of data elements available on all patients, supplemented by data available from other studies on various subsets of patients. We evaluated all available data elements which can be obtained from administrative sources (e.g., BC Cancer Registry) for accuracy, consistency, and completeness. We selected a set of data elements that met our quality standards. We deployed a pipeline that regularly performs quality checks on data elements against a set of rules that can be applied programmatically to validate the integrity, consistency, and logic between various elements before their integration. Only data that passed quality checks would be merged with a permanent clinical database; data that failed quality checks were further investigated with data stewards to determine sources of error. Clinical outcomes data from the BC Cancer Registry were de-identified before being merged with a permanent database hosted in REDCap, and updated quarterly.

To complement data available from the Registry, the second step of our process involved integrating clinical data obtained through clinical studies and held in silos. To ensure that data can be aggregated, compared, analyzed, shared and reused across studies, data standards were defined to resolve standardization discrepancies [44]. Unique data variables were aggregated from seven clinical studies to understand the breadth of the data in our clinical database. We created a standardized data dictionary with the goal of mapping data elements to the same data concepts across all clinical data collections in BC, these concepts in turn can be matched with a common data model OMOP-CDM [49] to maximize interoperability with external datasets.

### Data governance, ethics and standard operating procedures

Following standardization and aggregation of all our data sources, we developed a centralized governance model and defined protocols, standard operating procedures (SOPs) and policies governing data access, storage, protection, sharing and permissible use across OVCARE’s research community. To implement the governance framework, we designed, developed, tested and deployed the OVCARE Resource Portal (ORP). The portal was developed using Oracle APEX to provide an online interface for all internal research and collaborating teams to request for resources including biospecimen, clinical, molecular, imaging data as well as informatics and data analytics support.

## Supplementary Information

**Additional file 1.** Evaluation of identified biobanking library information management systems.

**Additional file 2.** OVCARE data commons: requirements identification and mapping desired biobanking features to solutions meeting the need.

## Data Availability

The LIMS survey data analyzed during the current study are available in Additional file 2: Tables S2–S12. The data are also publicly available on the websites (features section) of each surveyed LIMS (Additional file 1).

## Abbreviations

AI: Artificial Intelligence
APEX: Oracle Application Express
API: Application Programming Interface
CBGOU: Cheryl Brown Gynecological Cancers Outcomes Unit
CNAs: Copy Number Alterations
GUI: Graphical User Interface
HL7: Health Level Seven
HTTP: Hypertext Transfer Protocol
ICD: International Classification of Diseases
LIMS: Laboratory Information Management Systems
mRNA: Messenger RNA
MSK: Memorial Sloan Kettering Cancer Center
MySQL: My Structured Query Language
NGS: Next Generation Sequencing
OMOP-CDM: Observational Medical Outcomes Partnership-Common Data Model
ORP: OVCARE Resource Portal
OVCARE: Ovarian Cancer Research Program
PRO: Patient Reported Outcomes
REDCap: Research Electronic Data Capture
REST: Representational State Transfer
SOPs: Standard Operating Procedures
SQL: Structured Query Language
TARGET: Tumor Alterations Relevant for Genomics-Driven Therapy
TCGA: The Cancer Genome Atlas
TMA: Tissue Microarray

## References

[1] Vaught J (2016). Biobanking comes of age: the transition to biospecimen science. Annu Rev Pharmacol Toxicol.

[2] Vaught J, Kelly A, Hewitt R (2009). A review of international biobanks and networks: success factors and key benchmarks. Biopreserv Biobank.

[3] Eiseman E, Haga S (1999). Handbook of human tissue sources: a national resource of human tissue samples.

[4] Coppola L, Cianflone A, Grimaldi AM, Incoronato M, Bevilacqua P, Messina F (2019). Biobanking in health care: evolution and future directions. J Transl Med.

[5] Greenberg B, Christian J, Henry LM, Leavy M, Moore H. Biorepositories. Addendum to registries for evaluating patient outcomes: a user’s guide, third edition. Rockville (MD): Agency for Healthcare Research and Quality (US); 2018. (AHRQ Methods for Effective Health Care). http://www.ncbi.nlm.nih.gov/books/NBK493632/. Accessed 22 Jun 2021. 29671991

[6] Cortes A, Albers PK, Dendrou CA, Fugger L, McVean G (2020). Identifying cross-disease components of genetic risk across hospital data in the UK Biobank. Nat Genet.

[7] Harris JR, Burton P, Knoppers BM, Lindpaintner K, Bledsoe M, Brookes AJ (2012). Toward a roadmap in global biobanking for health. Eur J Hum Genet.

[8] Cole JB, Florez JC, Hirschhorn JN (2020). Comprehensive genomic analysis of dietary habits in UK Biobank identifies hundreds of genetic associations. Nat Commun.

[9] Collins FS, Varmus H (2015). A new initiative on precision medicine. N Engl J Med.

[10] Liu A, Pollard K, Karimi-Busheri F (2015). Biobanking for personalized medicine. Biobanking in the 21st Century.

[11] De Souza YG, Greenspan JS (2013). Biobanking past, present and future: responsibilities and benefits. AIDS.

[12] Uddin M, Wang Y, Woodbury-Smith M (2019). Artificial intelligence for precision medicine in neurodevelopmental disorders. Npj Digit Med.

[13] Jayshree P. Biobanking is changing the world. 2019. https://www.forbes.com/sites/cognitiveworld/2019/08/12/biobanking-is-changing-the-world/?sh=6cf563943792. Accessed 16 Aug 2020.

[14] Lee J-E (2018). Artificial intelligence in the future biobanking: current issues in the biobank and future possibilities of artificial intelligence. Biomed J Sci Tech Res..

[15] Kiehntopf M, Krawczak M (2011). Biobanking and international interoperability: samples. Hum Genet.

[16] Grossman RL, Heath A, Murphy M, Patterson M, Wells W (2016). A case for data commons: toward data science as a service. Comput Sci Eng.

[17] Jensen MA, Ferretti V, Grossman RL, Staudt LM (2017). The NCI Genomic Data Commons as an engine for precision medicine. Blood.

[18] Hinkson IV, Davidsen TM, Klemm JD, Chandramouliswaran I, Kerlavage AR, Kibbe WA (2017). A comprehensive infrastructure for big data in cancer research: accelerating cancer research and precision medicine. Front Cell Dev Biol.

[19] Köbel M, Rahimi K, Rambau PF, Naugler C, Le Page C, Meunier L (2016). An immunohistochemical algorithm for ovarian carcinoma typing. Int J Gynecol Pathol.

[20] Shah SP, Köbel M, Senz J, Morin RD, Clarke BA, Wiegand KC (2009). Mutation of *FOXL2* in granulosa-cell tumors of the ovary. N Engl J Med.

[21] Wiegand KC, Shah SP, Al-Agha OM, Zhao Y, Tse K, Zeng T (2010). *ARID1A* mutations in endometriosis-associated ovarian carcinomas. N Engl J Med.

[22] Errico A (2014). SMARCA4 mutated in SCCOHT. Nat Rev Clin Oncol.

[23] Wang YK, Bashashati A, Anglesio MS, Cochrane DR, Grewal DS, Ha G (2017). Genomic consequences of aberrant DNA repair mechanisms stratify ovarian cancer histotypes. Nat Genet.

[24] Talhouk A, McConechy MK, Leung S, Yang W, Lum A, Senz J (2017). Confirmation of ProMisE: a simple, genomics-based clinical classifier for endometrial cancer: molecular classification of EC. Cancer.

[25] Karnezis AN, Leung S, Magrill J, McConechy MK, Yang W, Chow C (2017). Evaluation of endometrial carcinoma prognostic immunohistochemistry markers in the context of molecular classification: additional IHC biomarkers in endometrial cancer in a new post-TCGA era. J Pathol Clin Res.

[26] Talhouk A, Hoang LN, McConechy MK, Nakonechny Q, Leo J, Cheng A (2016). Molecular classification of endometrial carcinoma on diagnostic specimens is highly concordant with final hysterectomy: earlier prognostic information to guide treatment. Gynecol Oncol.

[27] McAlpine JN, Leung SCY, Cheng A, Miller D, Talhouk A, Gilks CB (2017). Human papillomavirus (HPV)-independent vulvar squamous cell carcinoma has a worse prognosis than HPV-associated disease: a retrospective cohort study. Histopathology.

[28] Krishagni Solutions. OpenSpecimen. 2012. https://www.openspecimen.org. Accessed 17 Aug 2016.

[29] McIntosh LD, Sharma MK, Mulvihill D, Gupta S, Juehne A, George B (2015). caTissue suite to OpenSpecimen: developing an extensible, open source, web-based biobanking management system. J Biomed Inform.

[30] Krishagni Solutions. OpenSpecimen biobanking LIMS features. 2012. https://www.openspecimen.org/biobanking-lims-features/. Accessed 17 Aug 2016.

[31] Cerami E, Gao J, Dogrusoz U, Gross BE, Sumer SO, Aksoy BA (2012). The cBio Cancer Genomics Portal: an open platform for exploring multidimensional cancer genomics data. Cancer Discov.

[32] The Cancer Genome Atlas Research Network (2011). Integrated genomic analyses of ovarian carcinoma. Nature.

[33] Jonckheere N, Van Seuningen I (2018). Integrative analysis of the cancer genome atlas and cancer cell lines encyclopedia large-scale genomic databases: MUC4/MUC16/MUC20 signature is associated with poor survival in human carcinomas. J Transl Med.

[34] Cui X, Jing X, Yi Q, Long C, Tan B, Li X (2018). Systematic analysis of gene expression alterations and clinical outcomes of STAT3 in cancer. Oncotarget.

[35] The Cancer Genome Atlas Network (2012). Comprehensive molecular portraits of human breast tumours. Nature.

[36] Nagasawa S, Ikeda K, Horie-Inoue K, Sato S, Takeda S, Hasegawa K (2020). Identification of novel mutations of ovarian cancer-related genes from RNA-sequencing data for Japanese epithelial ovarian cancer patients. Endocr J.

[37] Gao J, Aksoy BA, Dogrusoz U, Dresdner G, Gross B, Sumer SO, et al. Integrative analysis of complex cancer genomics and clinical profiles using the cBioPortal. Sci Signal. 2013;6(269):pl1. 10.1126/scisignal.2004088 PMC4160307 23550210

[38] The Cancer Genome Atlas Program 2005. https://www.cancer.gov/about-nci/organization/ccg/research/structural-genomics/tcga. Accessed 12 Apr 2021.

[39] National Cancer Institute, Office of Cancer Genomics (OCG). TARGET: Therapeutically Applicable Research to Generate Effective Treatments. 2006. https://ocg.cancer.gov/programs/target. Accessed 15 Apr 2021.

[40] Harris PA, Taylor R, Thielke R, Payne J, Gonzalez N, Conde JG (2009). Research Electronic Data Capture (REDCap)—a metadata-driven methodology and workflow process for providing translational research informatics support. J Biomed Inform.

[41] Harris PA, Taylor R, Minor BL, Elliott V, Fernandez M, O’Neal L (2019). The REDCap consortium: Building an international community of software platform partners. J Biomed Inform.

[42] REDCap. Research Electronic Data Capture (REDCap). 2004. https://www.project-redcap.org. Accessed 15 Apr 2021.

[43] Oracle. Oracle Apex. 2019 https://apex.oracle.com/en/. Accessed 14 Apr 2021.

[44] Washington (DC). Sharing clinical research data: workshop summary. Washington (DC): National Academies Press (US); 2013. https://www.ncbi.nlm.nih.gov/books/NBK137818/. Accessed 26 Jun 2021. 23700647

[45] Seneviratne MG, Kahn MG, Hernandez-Boussard T (2019). Merging heterogeneous clinical data to enable knowledge discovery. Pac Symp Biocomput Pac Symp Biocomput.

[46] Huser V, Sastry C, Breymaier M, Idriss A, Cimino JJ (2015). Standardizing data exchange for clinical research protocols and case report forms: an assessment of the suitability of the Clinical Data Interchange Standards Consortium (CDISC) Operational Data Model (ODM). J Biomed Inform.

[47] De Maria MR, Di Sante G, Piro G, Carbone C, Tortora G, Boldrini L (2021). Translational research in the era of precision medicine: where we are and where we will go. J Pers Med.

[48] Tian Q, Price ND, Hood L (2012). Systems cancer medicine: towards realization of predictive, preventive, personalized and participatory (P4) medicine: Key Symposium: systems cancer medicine. J Intern Med.

[49] The Observational Health Data Sciences and Informatics (OHDSI). OMOP Common Data Model. https://www.ohdsi.org/data-standardization/the-common-data-model/. Accessed 2 Jul 2021.

