# Supplementary material for: From biobank and data silos into a data commons: convergence to support translational medicine
Source: J Transl Med. 2021 Dec 4;19:493. doi: 10.1186/s12967-021-03147-z (PMC8645144; doi:10.1186/s12967-021-03147-z)
Supplement: Supplementary file 1 — Additional file 1. Evaluation of identified biobanking library information management systems. [file 12967_2021_3147_MOESM1_ESM.docx]

**From biobank and data silos into a data commons: convergence to support translational medicine**

Rebecca Asiimwe^1,2^, Stephanie Lam^6,7^, Samuel Leung^1,3,7^, Shanzhao Wang ^3,7^, Rachel Wan^3^, Anna Tinker^4,5,7^, Jessica N. McAlpine^6,7^, Michelle Woo^7^, David Huntsman^1,3,6,7^, Aline Talhouk*^6,7^

^1^ Department of Molecular Oncology, BC Cancer Research Centre, 675 West 10th Avenue, Vancouver, British Columbia, Canada, V5Z 1L3

^2^ BC Children’s Hospital Research Institute, 938 West 28th Avenue, Vancouver, British Columbia, Canada, V5Z 4H4

^3^ Department of Pathology and Laboratory Medicine, Faculty of Medicine, University of British Columbia, 2211 Wesbrook Mall, Vancouver, British Columbia, Canada, V6T 2B5

^4^ Department of Medicine, Faculty of Medicine, Division of Medical Oncology, University of British Columbia, 2775 Laurel Street, Vancouver, British Columbia, Canada, V5Z 1M9

^5^ Division of Medical Oncology, BC Cancer, Vancouver Centre, 600 West 10th Avenue, Vancouver, BC V5Z 4E6, Canada

^6^ Department of Obstetrics and Gynecology, Faculty of Medicine, University of British Columbia, 1125 Howe Street, Vancouver, British Columbia, Canada, V6Z 2K8

^7^ OVCARE Research Program, BC Cancer - Vancouver, 600 West 10th Avenue, Vancouver, British Columbia, Canada, V5Z 4E6

*Corresponding author
†Current address:

Department of Obstetrics and Gynecology,

Faculty of Medicine, University of British Columbia,

5th Floor (593), VGH Research Pavilion

828 West 10th ave, Vancouver, BC   V5Z 1M9

604 875 4111 ext 21365

[a.talhouk@ubc.ca](mailto:a.talhouk@ubc.ca)

**Supplementary Materials: Additional File 1**

**Evaluation of identified biobanking LIMS**

Towards identifying an ideal solution that met OVCARE’s biobanking needs, we surveyed the biobanking environment and identified nine prominent biospecimen banking software solutions. In consideration were the following solutions including ATiM, the LIMS solution implemented at the time: 1) ATiM (1) **(Table S2)**, 2) OpenSpecimen (2) **(Table S3)**, 3) TissueMetrix (3) **(Table S4)**, 4) FreezerPro (4) **(Table S5)**, 5) Labmatrix (5) **(Table S6)**, 6) Caisis (6) **(Table S7)**, 7) CirraSpec (7) **(Table S8)**, 8) OBiBa (8) **(Table S9)**, 9) BiBBoX (9) **(Table S10)** and 10) BSI (10) **(Table S11)**. With reference to our requirements, each of these solutions were preliminarily assessed based on existing web documentation, reports and publications, scrutinizing each for the biobanking features and functionality provided, to include the length of time a biobank has been in existence, biobank user community, frequency of updates and upgrades, user support post installation and a probe on whether a biobank supports consent management, access control, multiple centers, cross-boundary studies/federated systems and integration with other systems, both local and remote. In some cases, evaluation involved running local instances of the biobanking software solution for which distributors provided trial runs. These trial runs were done to check if the biobanking solutions met OVCARE’s requirements and to check for: compatibility with the existing computing infrastructure, interoperability, acceptable data, privacy and security, access control mechanisms, ease of use and ability to alter base code and interfaces.

To further assess each LIMS’s fitness and ability to meet our key requirements, multiple meetings, interviews and live interactive demos were conducted with software providers. For example, demos for OpenSpecimen were held on October 21, 2016; FreezerPro on November 4, 2016; TissueMetrix on November 21, 2016 and BioFortis - Labmatrix on January 27, 2017. These vendor-based interactions and online demos were valuable in providing an in-depth review of the biobanking features and functionality of each LIMS, and provided a better exposition and elucidation of each biobank’s features than that obtained from online exhibitions and documentations. Besides our key biobanking features, continuous support, feasibility, pricing, subsequent software development to add missing key components and prospects to accommodate our future and changing needs were also discussed with software vendors.

The list of features **(Table S2 - S11)** obtained from the analysis of each solution was mapped to our requirements under the following main feature classifications: (1) Open-source software, (2) multi-tenancy, (3) data management, (4) administration, (5) security, (6) enrolment and consent, (7) storage and distribution, and (8) data querying and integration **(Table S12)**. The annual cost, year of release and last update, global adoption (the number of labs or institutions using the solution) were also features of interest in determining a suitable LIMS. All solutions were subsequently ranked based on the abundance of desired features per feature class. Based on these results, combined with information gathered from interviews, software demos and frequent vendor meetings, overall, OpenSpecimen overlapped with the majority of our biobanking requirements and was therefore considered for further evaluation.

**References**

1. Canadian Tissue Repository Network (CTRNet) - ATiM. https://www.ctrnet.ca/en/home/. Accessed 10 Aug 2016.

2. Krishagni Solutions. OpenSpecimen. https://www.openspecimen.org. Accessed 17 Aug 2016.

3. Artificial Intelligence in Medicine Inc. (AIM). TissueMetrix. https://www.aim.ca. Accessed 27 Oct 2016.

4. Brooks Automation. FreezerPro®. https://www.freezerpro.com. Accessed 13 Sep 2016.

5. BioFortis. Labmatrix. https://www.biofortis.com/labmatrix. Accessed 23 Aug 2016.

6. Memorial Sloan-Kettering Cancer Center (MSKCC). CAISIS. http://www.caisis.org. Accessed 2 Sep 2016.

7. TGen. Cirraspec®. https://www.tgen.org/research/shared-resources-services/cirraspec-biospecimen-management/. Accessed 18 Oct 2016.

8. Maelstrom Research, McGill University Health Centre (RI MUHC). OBiBa. https://www.obiba.org. Accessed 7 Oct 2016.

9. B3Africa. BIBBOX. http://bibbox.bbmri-eric.eu. Accessed 9 Sep 2016.

10. Information Management Services, Inc (IMS). Biological Specimen Inventory (BSI). https://www.bsisystems.com. Accessed 21 Sep 2016.
